# 110 Does Discharge to a Post-acute Facility Predispose Burn Patients to a Deterioration of Their Wounds?

**DOI:** 10.1093/jbcr/irac012.113

**Published:** 2022-03-23

**Authors:** Suzanne C Osborn, David Addison, Claudia Islas, Karen Richey, Kevin N Foster

**Affiliations:** Arizona Burn Center, Phoenix, Arizona; Arizona Burn Center Valleywise Health, Phoenix, Arizona; Arizona Burn Center Valleywise Health, Phoenix, Arizona; Arizona Burn Center Valleywise Health, Phoenix, Arizona; The Arizona Burn Center Valleywise Health, Phoenix, Arizona

## Abstract

**Introduction:**

The care of burn injuries requiring hospitalization is complex and highly specialized. In-hospital treatment results in discharging patients with stabilized, but unhealed wounds with the need for ongoing care. The expectation is that wounds continue to heal without complication. For patient who are unable to be discharged home, disposition is frequently dictated by finance and payor source. Post-acute facilities such as Skilled Nursing facilities (SNF), Acute Rehabilitation Centers (ARC) and Long-term Acute Care facilities (LTAC) are utilized. The purpose of this study was to evaluate the wound healing in patients being sent to SNF and ARC.

**Methods:**

A retrospective chart review of patients discharged to a SNF or ARC over a one-year period was performed. Photographic review was done comparing photos from the first clinic visit to those from hospital discharge. Wounds were designated as “Improved” (IM) or “Worsened/No Change” (W/NC) by a single burn surgeon.

**Results:**

Of the 963 charts reviewed, 719 patients suffered burn injuries, and of these 127 were discharged to post-acute facilities, 54 were either discharged to a LTAC or did not have photos available for review at their first clinic visit. Thus 73 were evaluable. The majority, 51% (n=37) worsened or showed no change (W/NC) and 49% (n=36) improved (IM). All patients returned to clinic within the first 2 weeks of discharge. There were no significance differences for age, gender, BMI, comorbidities, substance abuse, living situation, ICU days, ventilator days, length of stay, number of surgeries, payor source or facility type (SNF vs ARC). Mean TBSA was greater in IM 13.99% vs W/NC 8.31% (p=0.018). There were no significant differences between groups for mechanism, although IM was more likely to have suffered a flame/flash injury (n=20) and W/NC contact burns (n=21). A total of 30 different facilities were utilized for discharge.

**Conclusions:**

Despite having smaller burn injuries, the majority of patients worsened if discharged to a post-acute facility. No patient factors were identified that were associated with worsening/no change in wound status at the first clinic visit post discharge. Given the number of discharge facilities utilized, we were unable to analyze the relationship between specific facilities and outcomes. The magnitude of the problem warrants further investigation. A Quality Improvement project is being developed to further identify areas for intervention.

